# The next ‘pandemic playbook’ needs to prioritize the needs of children—and a clear roadmap for opening schools

**DOI:** 10.1017/ash.2023.154

**Published:** 2023-04-26

**Authors:** Westyn Branch-Elliman, Lloyd Fisher, Shira Doron

**Affiliations:** 1 Department of Medicine, VA Boston Healthcare System, Boston, Massachusetts; 2 Harvard Medical School, Boston, Massachusetts; 3 Reliant Medical Group, Worcester, Massachusetts; 4 Department of Pediatrics, University of Massachusetts Medical Center, Worcester, Massachusetts; 5 Tufts Medical Center, Boston, Massachusetts

## Abstract

The national influenza pandemic response plan includes short-term school closures as an infection mitigation measure, based on modeling data regarding the role of pediatric populations and schools as drivers of disease spread. Modeled estimates regarding the role of children and their in-school contacts as drivers of community transmission of endemic respiratory viruses were used in part to justify prolonged school closures throughout the United States. However, disease transmission models extrapolated from endemic pathogens to novel ones may underestimate the degree to which spread is driven by population immunity and overestimate the impact of school closures as a means of reducing child contacts, particularly in the longer-term. These errors, in turn, may have caused incorrect estimations about the potential benefits of closing schools on a society level while simultaneously failing to account for the significant harms of long-term educational disruption.

Pandemic response plans need to be updated to include nuances regarding drivers of transmission such as pathogen type, population immunity, and contact patterns, and disease severity in different groups. Expected duration of impact also needs to be considered, recognizing that effectiveness of different interventions, particularly those focused on limiting social interactions, are short-lived. Additionally, future iterations should include risk–benefit assessments. Interventions that are particularly harmful to certain groups, such as school closures are on children, should be de-emphasized and time limited. Finally, pandemic responses should include ongoing and continuous policy re-evaluation and should include a clear plan for de-implementation and de-escalation.

Throughout the pandemic, school-aged children have been subjected to some of the most stringent mitigation measures in all of society. Schools closed throughout the United States in March 2020. In large parts of the country, many did not open their doors to students again until the spring of 2021. Long past when masks were not required in most of society, mask mandates were maintained in schools. The reasons why these policies persisted for so long are multifactorial, but the long-standing postulate that children—and their day-to-day activities, such as attending school—drive respiratory pandemics certainly played a role. Experience during the coronavirus-19 disease (COVID-19) pandemic tells us that this mantra does not always apply and suggests that future pandemic policy planning needs nuance and a clear de-escalation plan based on prespecified goals of the interventions.

The 2006 National Strategy Implementation Plan for responding to influenza pandemics states the following:

“The clinical disease attack rate will be 30% in the overall population during the pandemic. Illness rates will be highest among school-aged children (∼40%) and decline with age.”^1^

The playbook goes on to suggest that, given the propensity of children to spread influenza, and the number of contacts children have in schools, short-term closures of elementary and secondary schools may be an effective policy for controlling the spread of influenza. The 2014 Updated Preparedness and Response Framework for Influenza Pandemics also includes “temporary” school closures as a potential community infection mitigation measure.^2^

A simplified theoretical model of viral transmission suggests that respiratory viral spread is determined in part by the number of susceptible contacts an infectious person encounters. Under this view of transmission, because children have many contacts in schools, and children are a relatively susceptible (ie, nonimmune) population, interventions that interrupt child-to-child interactions are likely to be highly effective not only for protecting pediatric populations but also for controlling community spread.^3^ The 2006 influenza pandemic playbook states the following:

“The clinical attack rates for seasonal and pandemic influenza are highest among children. Closure of schools and targeted vaccination of children have demonstrated efficacy in diminishing community influenza rates. Modeling supports school closure as an effective means of reducing overall attack rates within communities and suggests that the value of this intervention is maximized if school closure occurs early in the course of a community outbreak.”^1^

It is true that children have higher rates of endemic respiratory virus infections than adults; we see this with respiratory syncytial virus (RSV), influenza, and rhinovirus.^4–8^ But what if the paradigm—that children interacting in schools universally drive respiratory pandemics—is based on incorrect assumptions about the relative importance of in-school contact patterns for driving population spread, particularly in the longer-term?^1,6^ An alternate explanation for these well-recognized transmission patterns for endemic respiratory viruses is that children are always a more immune-susceptible population relative to adults (Fig. 1). Perhaps the reason endemic respiratory virus transmission appears to be driven by children is not because of their in-school contacts or behavior (although we acknowledge their respiratory hygiene may not be as robust as that of adults) but rather because children have not had time to acquire immunity to these endemic viruses to the extent adults have. Even during novel influenza pandemics,^9^ it appears that adult immunity from prior exposure to related influenza strains provides protection.

**Figure 1.**
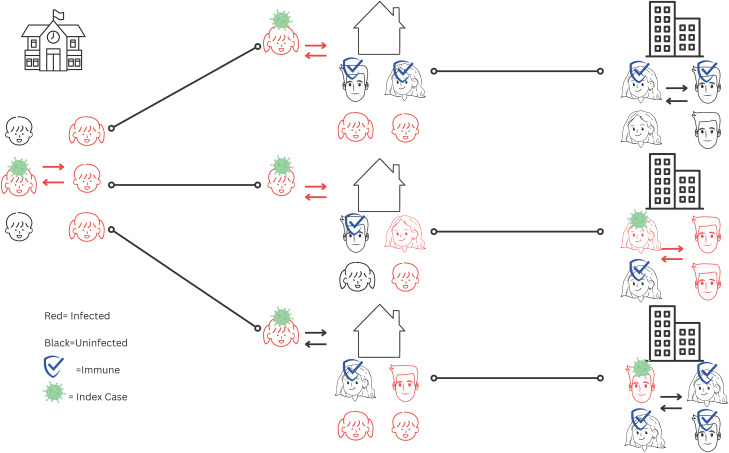
Theoretical model of endemic respiratory virus transmission: children and schools play a major role due to relatively low levels of immunity in pediatric versus adult populations. Theoretical scenario of transmission of an endemic respiratory virus in different settings (schools, home, places of work) with R_0_ = 3. Individuals with a virus icon indicate the index case. Red icons indicate an infected individual. Individuals with a shield icon are immune. In this scenario, children are a far more immune-susceptible population than adults, and thus the proportion of transmission that occurs in children and schools is high and substantial.

Even acknowledging that contact patterns are an important factor that determines spread of respiratory viruses in the population (which contributes to a second error resulting in lower real-world impact of school closures than is estimated from models) confers the assumption that school closures and other social distancing measures have a consistent effect on interrupting contact patterns.^10^ Limited data extrapolated from short-term (2-week) closures suggest that closing schools does reduce child-to-child contacts.^11^ However, subsequent studies evaluating longer periods found that although the number of contacts were reduced in the short-term, these reductions were short lived and childhood contacts quickly returned to baseline levels.^12^ Thus, as schools stay closed longer, theoretical benefits are reduced because children are interacting anyway, whereas harms increase (eg, learning disruptions, activity limitations, access to essential services).

If the alternate view is correct—that it is not children and schools per se primarily driving respiratory viral spread but rather the immune-susceptible population among the whole population—then we would not expect similar transmission dynamics for a novel respiratory virus, such as severe acute respiratory coronavirus virus 2 (SARS-CoV-2). We would expect adults and adolescents to contribute to spread at least as much as children. In fact, this is what we have seen; school-aged children do not appear to contribute substantially more to the population burden of SARS-CoV-2 than adolescents or adults, and disease transmission occurs in a variety of different settings (Fig. 2).^13–17^

**Figure 2.**
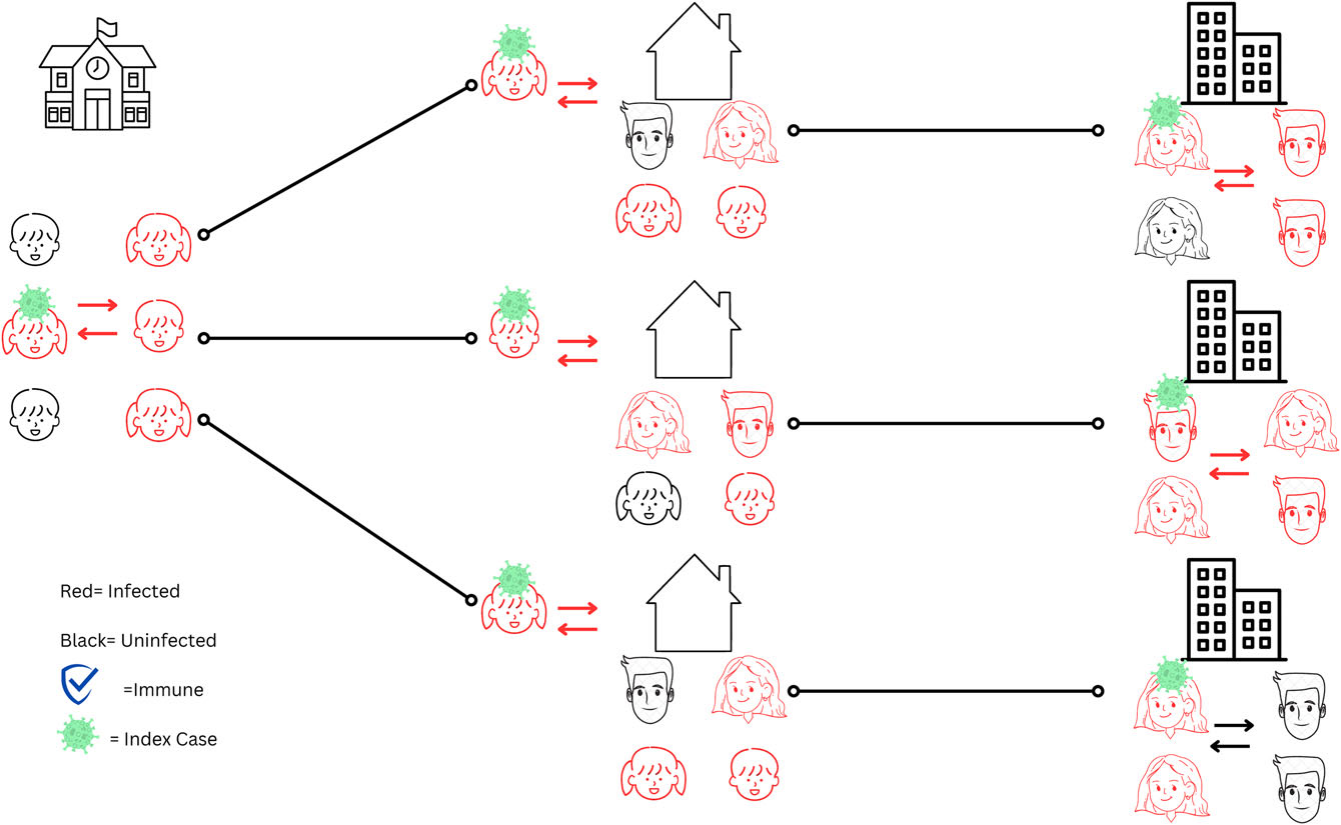
Theoretical model of novel respiratory virus transmission: transmission occurs in all types of settings and populations. Theoretical scenario of transmission of a novel respiratory virus in different settings (schools, home, places of work) with R_0_ = 3. Individuals with a virus icon indicate the index case. Red icons indicate an infected individual. Individuals with a shield icon are immune. In this scenario, all populations are susceptible, and transmission occurs in all settings and populations, with a relatively lower proportion of the total number of cases attributable to pediatric populations and schools.

Population immunity against coronaviruses is more complex than that of influenza. Children are more likely to have asymptomatic carrier states than adults.^18,19^ Although there is some controversy about the role asymptomatic cases play in population spread, studies suggest transmission is more common from symptomatic and presymptomatic patients than truly asymptomatic ones.^20^ Furthermore, due to limited existing immunity across all ages, even if children have similar transmission potential as adults, the proportion of transmission attributable to children in schools is lower. Additionally, unlike with influenza, there is a steep age gradient for disease severity, and children are at lower risk of severe disease than adults.^21^ Considering children as a less at-risk population compared to adults and adolescents, even with similar transmission potential, upends infectious diseases models, on which coronavirus pandemic responses have been based and prolonged school closures justified. Real-world implementation challenges, specifically short-lived adherence to social distancing measures,^22^ further diminish the expected benefits of school closures relative to estimates generated in theoretical infectious disease transmission models.

The relatively powerful role of whole population immunity in driving SARS-CoV-2 transmission and community disease burden relative to the short-term impact of disrupting contact patterns of school-aged children suggests that updates are needed to how we think about respiratory pathogen pandemics and how we respond to them. Specific mitigation policies designed to reduce spread driven by children—most notably school closure—would be expected to have a substantially lower impact on community case rates and population outcomes than would be expected based on influenza transmission models. Real-world experience with children and estimates of the effectiveness of school closures during the COVID-19 pandemic validate the hypothesis that other factors, particularly a lack of immunity, was the primary driver of community case rates.^23,24^ Failure to recognize barriers to the sustainability of school closures as a means for reducing contacts also likely contributed to overestimates of benefit in theoretical disease models. A confluence of these factors likely explains the minimal impact long-term school closures had on transmission in the real world.

Notably, the pandemic response plan only recommends consideration of short-term closures, not long-term ones lasting months, as occurred in the United States and some countries across the world. But we have also learned that once schools are closed, the path to reopening them is not straightforward, particularly without a clear roadmap for the process to do so and if schools are not specifically prioritized during the de-escalation process. The harmful effects of school closures on this generation of children continue to be elucidated, but evidence suggests substantial long-term negative impacts.^25^ Thus, it is imperative that before any community-level mitigation measures are implemented, there should be a clear plan for de-escalation with goals and duration of the measure specified upfront. Deimplementation strategies should be prespecified. Additional research is needed to determine the effectiveness of other sustainable infection mitigation strategies, such as ventilation upgrades and high-efficiency particulate air (HEPA) filtration systems, in school settings. The ongoing cluster-randomized controlled trial evaluating the utility of HEPA filters will provide important information to guide future policies.^26^ Other infection mitigation measures, such as testing programs and masking policies, may be useful temporary measures for safely maintaining in-person learning, especially for pathogens for which there is limited population immunity. However, currently available data on the impact of mask mandates in school settings have been inconclusive; different studies have variable results and come to different conclusions.^27,28^ The theoretical benefits of interventions that require high levels of daily adherence to be effective need to be weighed against real-world feasibility and implementation challenges and harms of the interventions, including learning and communication disruptions.

Early and temporary school closures during spring of 2020 were probably inevitable and may have been justified while we gained a better understanding of the transmission mechanisms of SARS-CoV-2, and it is likely that school closures during this early phase of the pandemic did reduce contacts between children. However, over a relatively short period of time, child contacts increased, even as schools remained closed.^12^

Although early closures may have been unavoidable, the lack of a well-delineated plan for prioritizing reopening schools coupled with a strong message in support of in-person educational opportunities from federal public health authorities was not. The H1N1 pandemic arrived in the spring of 2009. That winter, a clear message was disseminated by the Centers for Disease Control and Prevention:

“Based on the experience and knowledge gained in jurisdictions that had large outbreaks in spring 2009, the potential benefits of preemptively dismissing students from school are often outweighed by negative consequences, including students being left home alone, health workers missing shifts when they must stay home with their children, students missing meals, and interruption of students’ education.”^29^

A similar approach should have been followed for COVID-19. Population immunity and pandemic politics are both complex phenomena, which means that advance planning is both challenging and fraught with uncertainty. It is impossible to predict the future, but the way to address uncertainty is to embrace and acknowledge it. Policy makers should be transparent with the public about the idea that recommendations will change based on new evidence and new advancements. Updating public health recommendations based on new evidence and changing conditions is not admitting error. Rather, evolving recommendations as we learn more is the best policy.^30^

During the early national response to the COVID-19 pandemic, extrapolated assumptions about the role of elementary and secondary school-aged children in driving spread of respiratory infections were applied to this novel pathogen and informed public health policy. But SARS-CoV-2 and influenza have critical distinctions, and future pandemic pathogens are likely to be different from both. Therefore, we need a more nuanced pandemic policy response plan that includes a clear statement of goals, transmission potential, risk–benefit considerations, realistic expectations about duration of impact of interventions, and a prespecified de-escalation plan. Fear-based messaging should be avoided, and rapid return to normalcy should be specifically prioritized and integrated into planning.

We need different playbooks and plans that account for susceptibility to infection, severity of disease in the population being primarily targeted for the intervention, transmission potential, and the expected duration of impacts of an intervention in the real world. Pandemic policy re-evaluation should be continuous and ongoing, as proposed in the Learning Health Policy System framework.^30^ Ongoing pandemic policy evaluations should include risk–benefit analysis to prioritize different infection mitigation strategies and to help determine when and how interventions are lifted. In adopting policy, children need to be prioritized. Even in situations in which children are more susceptible to severe disease, infection mitigation interventions that target and harm the long-term health and well-being of children, such as closure of schools, should be still de-emphasized, limited in duration, and weighed against the substantial harms of such policies. Future pandemic plans need to specify that schools are last to close and first to open.
